# Prevalent Hallucinations during Medical Internships: Phantom Vibration and Ringing Syndromes

**DOI:** 10.1371/journal.pone.0065152

**Published:** 2013-06-10

**Authors:** Yu-Hsuan Lin, Sheng-Hsuan Lin, Peng Li, Wei-Lieh Huang, Ching-Yen Chen

**Affiliations:** 1 Department of Psychiatry, National Taiwan University Hospital, Yun-Lin Branch, Yunlin, Taiwan; 2 Department of Epidemiology, Harvard School of Public Health, Boston, Massachusetts, United States of America; 3 School of Medicine, Chang Gung University, Taoyuan, Taiwan; 4 Department of Psychiatry, Chang Gung Memorial Hospital at Lin-Kou, Taoyuan, Taiwan; UNLV, United States of America

## Abstract

**Background:**

Phantom vibration syndrome is a type of hallucination reported among mobile phone users in the general population. Another similar perception, phantom ringing syndrome, has not been previously described in the medical literature.

**Methods:**

A prospective longitudinal study of 74 medical interns (46 males, 28 females; mean age, 24.8±1.2 years) was conducted using repeated investigations of the prevalence and associated factors of phantom vibration and ringing. The accompanying symptoms of anxiety and depression were evaluated with the Beck Anxiety and Depression Inventories before the internship began, and again at the third, sixth, and twelfth internship months, and two weeks after the internship ended.

**Results:**

The baseline prevalence of phantom vibration was 78.1%, which increased to 95.9% and 93.2% in the third and sixth internship months. The prevalence returned to 80.8% at the twelfth month and decreased to 50.0% 2 weeks after the internship ended. The baseline prevalence of phantom ringing was 27.4%, which increased to 84.9%, 87.7%, and 86.3% in the third, sixth, and twelfth internship months, respectively. This returned to 54.2% two weeks after the internship ended. The anxiety and depression scores also increased during the internship, and returned to baseline two weeks after the internship. There was no significant correlation between phantom vibration/ringing and symptoms of anxiety or depression. The incidence of both phantom vibration and ringing syndromes significantly increased during the internship, and subsequent recovery.

**Conclusion:**

This study suggests that phantom vibration and ringing might be entities that are independent of anxiety or depression during evaluation of stress-associated experiences during medical internships.

## Introduction

Phantom vibration syndrome, an intermittent perception that a mobile phone is vibrating when it is not [1], may be a prevalent hallucination in the general population. The only previous cross-sectional study of phantom vibration syndrome estimated that 68% of medical staff members had experienced this phenomenon [1]. A similar perception, phantom ringing syndrome, an intermittent perception that a mobile phone is ringing when it is not, has not been described in the medical literature. Thus, its prevalence and associated factors remain unknown.

These two “syndromes” might share common associated factors and underlying mechanisms. Furthermore, phantom ringing, an auditory hallucination, warrants further study because it is more similar to the psychopathology of a major mental illness than is phantom vibration. Hallucinations are aberrant perceptions in the absence of a stimulus; thus, phantom vibration and phantom ringing could be defined as tactile and auditory hallucinations [2]. Compared with tactile and other hallucinations, auditory hallucinations are common in psychotic disorders such as schizophrenia [3], although they are typically related to verbal voices rather than to nonverbal auditory hallucinations, such as phantom ringing of a mobile phone. The extremely high prevalence of phantom vibration reported by medical staff members attests to the fact that normal brain mechanisms are at work. In a community sample of young adults, individuals with higher levels of psychological distress also reported higher frequencies of hallucination-like experiences [4]. Such auditory perceptual distortions may represent a state of general vulnerability in the general population [5].

A previous cross-sectional study identified four factors that were independently associated with phantom vibration: being a medical student or resident, the number of hours that the phone was carried, more frequent use of the phone in vibration mode, and carrying the device in a breast pocket [1]. The first two factors can be attributed to the long work hours and stressful workload of medical students and residents. Thus, we designed a prospective cohort study to investigate the factors associated with phantom vibration and phantom ringing hallucinations during the medical internship year. We chose this particular setting because a medical internship provides a rare instance in which the onset of a major stressor can be predicted for a defined population. In addition, the age, lifestyle, and educational background of medical interns are similar. Furthermore, in Taiwan, medical interns have a short period of free time after their internship, so the internship provides a good stress exposure model with which to study the relationship between stress and hallucinations.

The specific aims of the present prospective longitudinal study were: (1) to assess the prevalence of phantom vibration and phantom ringing throughout the internship; (2) to identify the factors associated with the development of phantom vibration and phantom ringing; and (3) to investigate the baseline personality traits, stress during internship, the accompanied depression and anxiety levels, and their correlations with phantom vibration and phantom ringing.

## Methods

### Participants

We recruited 74 medical interns (46 males and 28 females, with a mean age of 24.8 years; standard deviation [SD], 1.2, range: 23–29) of 136 (males, 99) medical interns who were trained at Chang Gung Memorial Hospital for one year. One female intern was lost to follow-up due to non-academic leave. The participants were volunteers recruited from the seventh grade of a medical college student population. All participants were healthy and none had any mental illnesses. The assessments took place prior to and during the third, sixth, and twelfth months of their internship. The assessments also took place 2 weeks after their internship, and none of the participants had to work during those 2 weeks. During their internship, the participants worked approximately 86.7 hours a week, including 33.5 consecutive work hours and an average of 10 on-call duties monthly, which they did not have before the internship [6]. The results of a previous study also suggested reduced cardiac parasympathetic modulation and increased depressive symptoms in the male group during medical internship [7]. Participants completed a baseline survey 1 to 2 months prior to beginning their internships. The survey assessed general demographic factors (age and gender), and included a phantom vibration and phantom ringing questionnaire, in addition to the following psychological measures: (1) the Tridimensional Personality Questionnaire (TPQ), (2) the Beck Depression Inventory (BDI), and (3) the Beck Anxiety Inventory (BAI). The BDI and BAI are widely used self-administered instruments for detecting symptoms of depression and anxiety. The participants were assessed using the phantom vibration and phantom ringing questionnaire, the BAI, and the BDI at the third, sixth, and twelfth months of their internship year and two weeks after their internship was completed. All participants provided informed written consent and were given 1000 new Taiwan dollars (NTD) after finishing the study. The study ran from May 2011 through June 2012. The study protocol was approved by the Ethics Committee of Chang Gung Memorial Hospital.

### Measurements

#### The phantom vibration and ringing questionnaire

To avoid biasing the respondents, the phantom vibration and ringing questionnaire simply stated, “We are asking you to participate in a research study survey about cell phones, because in your job you carry one.” The questionnaire also asked if the respondent had experienced phantom vibration and phantom ringing during the 3-month period, as well as potential factors associated with phantom vibration that were documented in the previous cross-sectional study [2]. For example, the questionnaire asked whether the device was used in the vibration or ringing mode, and where it was worn. Those who reported phantom vibration or phantom ringing were also asked how bothersome these events were.

#### The tridimensional personality questionnaire

One of the more recent tools used to investigate personality traits is the TPQ, which is based on Cloninger’s psychobiological model of personality [8]. The questionnaire consists of three genetically independent dimensions: novelty seeking (NS), harm avoidance (HA), and reward dependence (RD). The Chinese version of the TPQ contains 100 “true” or “false” items converting three NS, HA, and RD dimensions, with four subscales for each. Chen et al. translated the TPQ into Chinese in 2002 [9], and this version of the questionnaire was used in our study.

#### The beck depression and anxiety inventories

The BDI is a widely used self-administered instrument for detecting depression and anxiety symptoms and for screening subjects with possible clinical depression [10]. This study used the Chinese version of the BDI, which includes 21 statements rated from 0 to 3. In this version, the scores are interpreted as: 0−13 = normal; 14−19 = mild depression; 20−28 = moderate depression; and 29−63 = severe depression [11].

The BAI is a 21-question multiple-choice self-report inventory used for measuring the severity of an individual's anxiety. This questionnaire is designed for individuals aged 12 years and older, and is composed of items relating to the symptoms of anxiety [12]. In this study, we used the Chinese version of the BAI, which includes 21 statements rated on a scale from 0 to 3. In this version, anxiety is scored in the following manner: 0−7 = minimal anxiety; 8−15 = mild anxiety; 16−25 = moderate anxiety; and 26−63 = severe anxiety [13].

### Statistical Analysis

Generalized estimating equation (GEE) methods were used to examine the effects of the internship at the third, sixth, and twelfth months and 2 weeks after the internship ended with regard to the prevalence of phantom vibration/ringing and BAI and BDI scores. The GEE is a generalized linear model estimation method for longitudinal data in which the within-group correlation can be specified. It fits a population-averaged model. Thus, the method accounted for the repeated measures obtained from each participant at different stages of the internship [14].

Four predictive models were designed to examine the predictors of phantom vibration/ringing and anxiety/depression status. Since the primary outcomes are binary variables in the first two models of phantom vibration/ringing, the Bernoulli distribution of the outcome variables conditioning on all predictors and the logistic link function were assumed, while the normal distribution was assumed in the last two predictive models of anxiety and depression, in which both outcome of interest were continuous variables. For the correlation structure, the unsaturated (fully parameterized clusters) log odds ratio matrix and the unstructured variance-covariance matrix were used for the model of phantom vibration/ringing and the model of anxiety/depression, separately.

The effects on phantom vibration and phantom ringing occurrence mediated by the factors of gender, TPQ, BAI, and BDI scores, the physical location in which the interns carried their cell phones, and vibration mode usage were examined in the model. Because all of the interns constantly used their cell phones in the ringing mode, we examined the effect of vibration mode usage on both phantom vibration and phantom ringing prevalence. We also investigated whether the TPQ scores differed between interns with or without phantom vibration/phantom ringing experiences prior to their internship, using the independent t-test. The effects of all these predictors were expressed by prevalence ratio, the exponential of regression coefficients in the model of phantom vibration/ringing shown in Table 1. In the models of anxiety and depression, only the effect of the internship, TPQ scores, and gender were involved. The effects of all the previous predictors were expressed by the regression coefficients, as shown in Table 2.

**Table 1:** Model of predictors for experiencing phantom vibration/ringing.

Characteristic		Prevalence ratio of phantom vibration				Prevalence ratio of phantom ringing			
		Estimate	95% CI		*P*- value	Estimate	95% CI		*P-* value
Intercept		0.69	0.13	3.67		0.21	0.04	1.06	
Before internship		1.00 (reference)				1.00 (reference)			
Internship	3rd month	8.74*	2.36	32.45	0.0012	15.16*	5.87	39.14	<0.0001
	6th month	5.17*	1.55	17.31	0.0076	21.71*	7.97	59.19	<0.0001
	12th month	1.30	0.52	3.23	0.5687	16.99*	6.45	44.75	<0.00001
After internship		0.34*	0.16	0.75	0.0069	2.81*	1.29	6.13	0.0092
Device location	Breast pocket	1.00 (reference)				1.00 (reference)			
	Belt	1.48	0.35	6.32	0.5992	4.35	0.54	35.07	0.1676
	Side pocket of white coat	1.31	0.73	2.34	0.3596	0.62	0.34	1.11	0.1073
	Back pocket	0.42	0.11	1.57	0.1982	1.06	0.33	3.38	0.9237
	Hanging	0.55	0.18	1.67	0.2955	0.49	0.20	1.24	0.1325
	Side pocket of trousers	4.41	0.41	47.49	0.221	0.47	0.08	2.93	0.4221
Vibration mode	Not used	1.00 (reference)				1.00 (reference)			
	Used	2.41*	1.32	4.38	0.004	1.61	0.91	2.87	0.1041
Gender (male vs. female)		1.13	0.61	2.10	0.6917	1.14	0.60	2.16	0.6995
Anxiety		0.99	0.94	1.04	0.7814	0.98	0.94	1.03	0.4436
Depression		1.03	0.98	1.08	0.1999	1.01	0.96	1.05	0.7397
Personality	NS	0.98	0.91	1.06	0.6465	1.02	0.97	1.08	0.4196
	HA	1.04	0.99	1.09	0.1383	1.02	0.97	1.06	0.4851
	RD	1.00	0.92	1.08	0.949	0.99	0.91	1.08	0.8543

Key: CI, confidence interval; NS, novelty seeking; HA, harm avoidance; RD, reward dependence, according to the Tridimensional Personality Questionnaire; *significant changes from baseline (P<0.05).

**Table 2:** Model of predictors for anxiety and depression scores.

Characteristic		Regression coefficient for depression				Regression coefficient for anxiety			
		Estimate	95% CI		P value	Estimate	95% CI		P value
Intercept		5.11	−0.41	10.64	0.0742	−2.15	−7.37	3.06	0.4214
Before internship		1.00 (reference)				1.00 (reference)			
Internship	3rd month	6.55*	4.64	8.46	<0.0001	3.57*	1.50	5.65	0.0009
	6th month	5.84*	3.64	8.03	<0.0001	3.81*	1.52	6.10	0.0013
	12th month	6.64*	4.37	8.92	<0.0001	3.67*	1.34	6.00	0.0022
After internship		0.56	−1.74	2.87	0.6331	0.40	−1.95	2.74	0.7398
Gender (male vs. female)		−1.28	−3.23	0.67	0.2029	−1.48	−3.30	0.35	0.1172
Personality	NS	−0.01	−0.25	0.22	0.9224	−0.15	−0.37	0.07	0.1873
	HA	0.34*	0.20	0.49	<0.0001	0.31*	0.17	0.44	<0.0001
	RD	0.01	−0.27	0.29	0.9543	0.42*	0.16	0.68	0.0025

Key: CI, confidence interval; NS, novelty seeking; HA, harm avoidance; RD, reward dependence, according to the Tridimensional Personality Questionnaire; *significant changes from baseline (P<0.05).

All analyses were performed using Statistical Analysis System (SAS) 9.2 software (SAS Institute, Cary, NC, USA), PROC GENMOD and PROC MIXED. Statistical significance was considered at the level of P<0.05.

## Results

**Figure 1:**
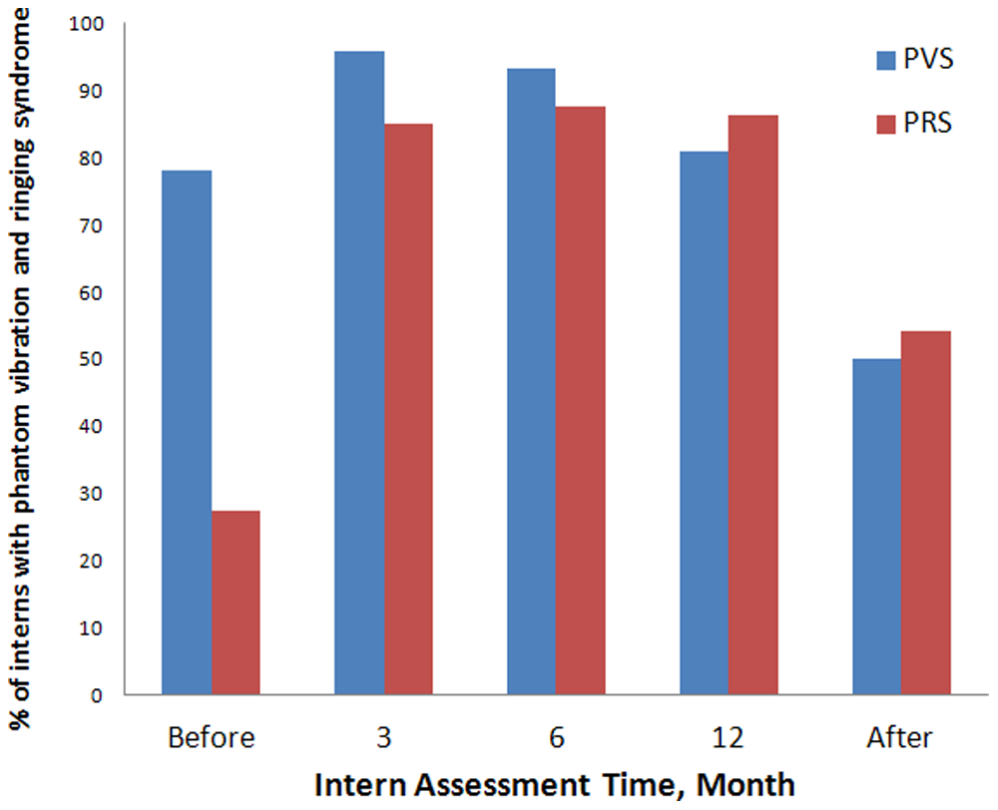
The proportion of interns who reported phantom vibration syndrome (PVS) and phantom ringing syndrome (PRS) before and after the internship and at months 3, 6, and 12 of the internship.

As shown in Figure 1, the baseline prevalence of phantom vibration was 78.1% prior to the internship. This significantly increased to 95.9% and 93.2% at the third and sixth internship months, then declined to 80.8% at the twelfth month and significantly decreased below baseline levels, to 50.0%, 2 weeks after the internship ended. The baseline phantom ringing prevalence was 27.4%, which significantly increased to 84.9%, 87.7%, and 86.3% at the third, sixth, and twelfth internship months, respectively. The phantom ringing prevalence decreased to 54.2% 2 weeks after the internship ended, and remained significantly higher than at baseline.

We used the generalized estimating equation (GEE) model to examine the predictive factors associated with phantom vibration and phantom ringing (Table 1). We confirmed that the odds ratio of the hallucination prevalence was significantly changed during the internship, as described before. The use of vibration mode increased the odds of phantom vibration but not the odds of phantom ringing. The factors of gender, the location in which the interns carried their phones, and the TPQ, BAI, and BDI scores were not related to the prevalence of phantom vibration or phantom ringing.

We also examined the relationship between personality and baseline phantom vibration/phantom ringing prevalence before the internship year. Interns with phantom ringing had significantly higher scores of NS than those without phantom ringing. However, there was no difference in HA and RD and no indices of TPQ differed between participants that did or did not experience phantom vibration and phantom ringing during the internship.

**Figure 2:**
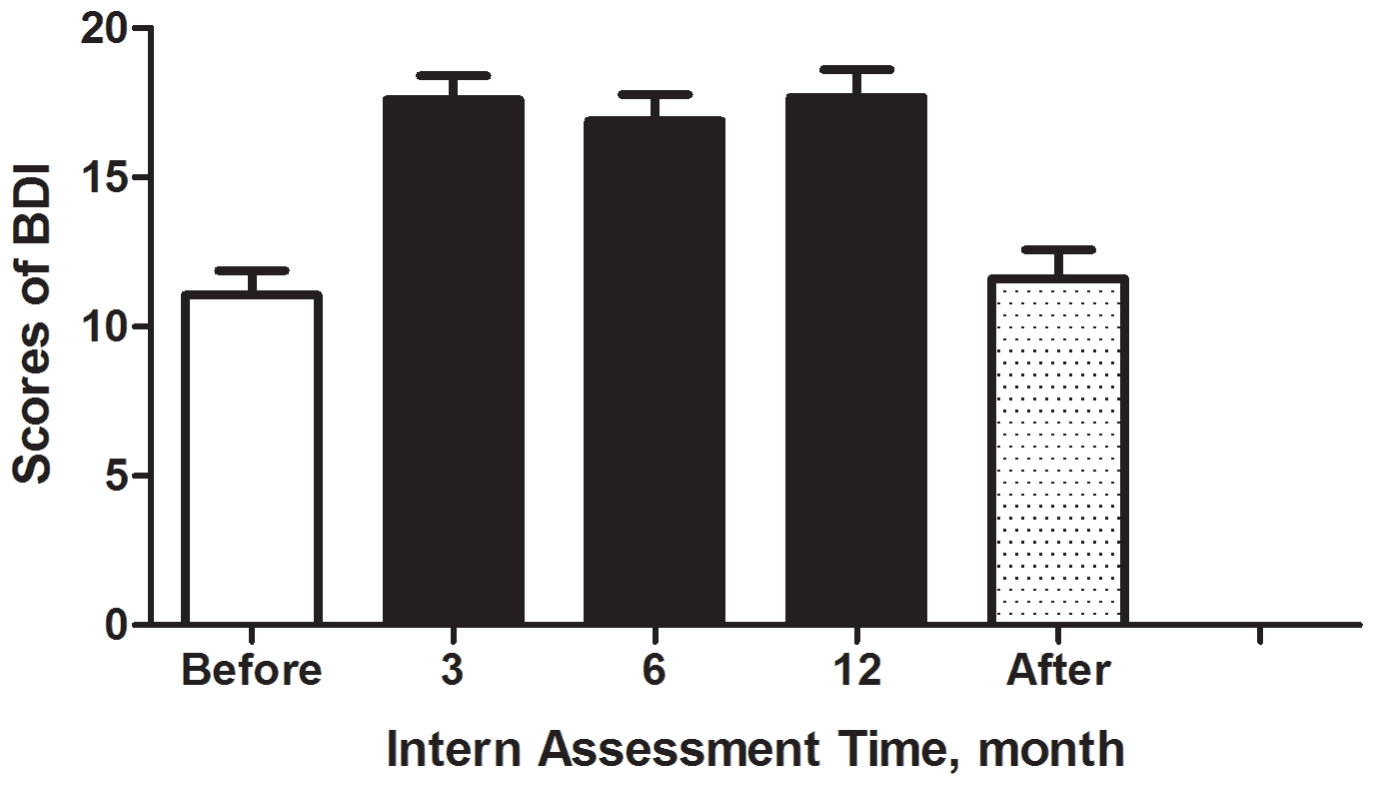
Beck Depression Inventory (BDI) scores before and after internship and at months 3, 6, and 12 of the internship.

**Figure 3:**
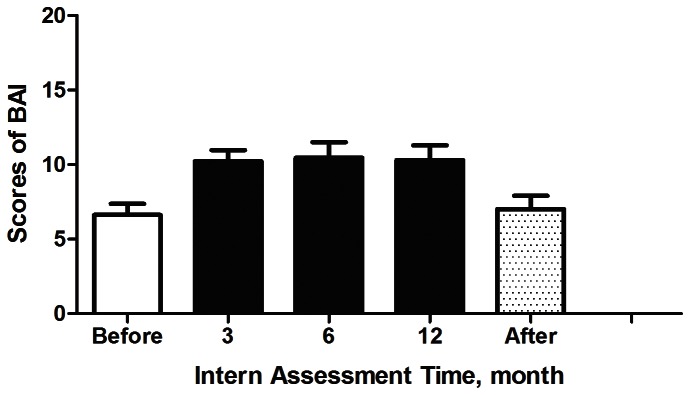
Beck Anxiety Inventory (BAI) scores before and after the internship and at months 3, 6, and of the internship.

Figure 2 shows that the depression scores increased from minimal depression (11.1±6.9) to mild depression (17.6±6.9, 16.9±7.5, 17.7±7.8) during the third, sixth, and twelfth internship months, respectively, and then returned to baseline values 2 weeks after the internship ended (11.6±8.2). Similarly, anxiety scores increased from minimal anxiety (6.6±6.4) to mild anxiety (10.2±6.4, 10.5±8.9, 10.3±8.4, respectively) during the internship, and then returned to baseline values 2 weeks after the internship (7.0±7.5) (Figure 3). Table 2 demonstrates that the presence of higher HA and RD values resulted in increased anxiety scores, and only higher HA measurements led to increased depression scores. Neither depression nor anxiety was related to gender.

## Discussion

The results of this longitudinal survey are consistent with those of a previous cross-sectional study [1] with regard to the use of vibration mode as an associated factor and the absence of gender differences in the prevalence of phantom vibration. The time course of the present study enhances previous findings demonstrating that 41% of individuals began to experience phantom vibration after one to five months of carrying a cell phone. Moreover, we clarified the role of workload in the development of phantom vibration and phantom ringing, which was impossible to estimate due to the highly co-linear nature of the previous sample.

Another hallucination, phantom ringing, was investigated for the first time in the present study. The prevalence of phantom ringing was much lower than that of phantom vibration prior to the internship and it increased approximately threefold during the internship, and then partially returned to baseline after the internship ended. However, we cannot examine whether ringing mode was associated with phantom ringing because all of the participants in this study constantly used the ringing mode. Our study could not replicate the results indicating that carrying a cell phone in the breast pocket is a risk factor for phantom ringing or phantom vibration. In the previous cross-sectional study, all eight medical staff members who carried a mobile phone in their breast pockets experienced phantom vibration, but the small sample size might have included different types of medical staff members, whereas all subjects in this study were medical interns.

The prevalence of phantom vibration before the internship (78%) was similar to that reported for medical students (90%) and resident physicians (80%) in previous study [1], although the prevalence significantly increased to 93−96% during the first six months of the internship. The simultaneous elevated prevalence of phantom ringing and anxiety and depression scores underscores the important role of stress during the internship. These findings provide further evidence for the contribution of non-genetic factors to the etiology of psychotic symptoms [15] [16].

Interestingly, no significant correlation exists between phantom vibration/phantom ringing and anxiety or depression. The anxiety and depression scores obtained 2 weeks after the internship ended were nearly equal to those reported before the internship began; this was not true for the prevalence of phantom vibration and phantom ringing. The baseline personality traits predicted most of the anxiety or depression during the internship but they did not predict the hallucinations that occurred during this period. It is conceivable that the prevalence of phantom vibration 2 weeks after the internship ended is lower than the baseline prevalence because interns who were not working after their internship were probably less stressed than they were as medical students before the internship began.

These findings implied that phantom vibration and phantom ringing are entities that are independent of anxiety or depression, and that internship-related stress might have simultaneously increased the interns’ anxiety, depression, and hallucinations.

Although hallucinations are sometimes pathological, they often occur in normal individuals. Previous studies have shown that an increased top-down influence of imagery on perception works together with deficient reality monitoring to generate hallucinatory experiences [17]. In our study we discovered that hallucinatory experiences are extremely common in some circumstances, and the results indicated that normal brain mechanisms are at work. These experiences may be determined by top-down psychological factors such as vigilance, which are aggravated by stress.

Two hypotheses may explain this finding. First, there is a high degree of expectancy for such experiences, which could amplify top-down processing, producing these false precepts even in the absence of stress. This mechanism may have occurred during the internship because an emotionally salient stimulus, for example, bereavement, experienced repeatedly in an aroused state, leads to a hypervigilant state in which a similar perception is experienced in the absence of the stimulus and lowers the threshold for hallucination [18]. Second, these are repetitive memories of previous real experiences that might predispose to hallucinated recurrences through some sort of conditioning. And the fact that an intern, who is more likely to require urgent attention, has to be on high alert for such experiences is likely to play a role. A correlation between vibration mode and phantom vibration in this study also supported this viewpoint. Furthermore, there might be more variation in the type and volumes of the ringing mode than vibration mode alone, which is relatively uniform. The top-down process was less amplified in the development of phantom ringing than was vibration before the internship began. That is, phantom vibration was more common at baseline than was phantom ringing.

Our study provides a model of stress-induced psychotic symptoms that is superior to the model of childhood trauma and psychotic illnesses [19], as we demonstrated that these hallucinations are reversible after the internship ended. Neurodevelopmental changes associated with the hypothalamic-pituitary-adrenal (HPA) axis have been investigated as mechanisms of auditory hallucination among children who have experienced trauma [20] and posttraumatic stress disorder [21]–[23]. Our previous study also showed that the heavy workload during an internship resulted in long- and short-term alterations of autonomic nervous system modulation [6].

The two major findings in relation to personality characteristics are: (1) interns with phantom ringing had significantly higher scores of novelty seeking than did those without phantom ringing, and (2) higher HA measurements led to increased depression scores. Cloninger's tridimensional personality theory offers three independent “temperament” dimensions – novelty seeking, harm avoidance, and reward dependence [8]. In previous studies, the relationship between harm avoidance and anxiety/depression has been well established [24], and novelty seeking has been associated with the dopamine system. Based on the hypothesis that phantom vibration and phantom ringing are transient, abnormal novelty rewards, the present study further corresponded to the well-known overlapping circuits that mediate stress responses, emotional learning, and reward processing [25]. Additionally, the increased NS behavior in medical interns who experienced phantom ringing before the internship partially explains the formulation of this auditory hallucination, although stress plays the most important role. The dopamine system, with a nearly universally accepted central role in psychotic disorders, mediates the experience of novelty and the acquisition of appropriate motivational salience [26]–[28]. In the processing of anticipation, people with higher novelty seeking scores have higher activity in the frontal cortex compared with others [29]. Our findings may support that hallucinatory experiences can be a presentation of anticipation, which is correlated to the novelty seeking personality [29].

Several limitations to our study should be noted. First, 78% and 27% of participants reported phantom vibration and phantom ringing, respectively, before the internship began. In addition to personality characteristics (i.e., novelty seeking), increased predisposing factors such as the cognitive approach [3] of phantom vibration and phantom ringing in these populations should be explored. Besides, the TPQ did not include questions about the hallucinatory trait. Instead, the dimension “psychoticism” in the Eysenck Personality Questionnaire [30] might have helped examine the hallucinatory trait of phantom vibration/ringing. Second, all investigations were self-reported, and a more objective method is required to understand the underlying mechanism. For example, the well-validated neurophysiological measures in event-related potentials, such as prepulse, may reflect the deficit in “gating out” of particular sensory information in subjects with these types of hallucinations [31]. The measurement of autonomic modulation, such as heart rate variability [32], may also make it possible to examine the role of the HPA axis in the development of these hallucinations. Third, the frequency or severity of these hallucinations could be applied in a future dimensional approach analysis rather than with the current categorical approach. The role of negative emotions in hallucinatory experiences may exert an influence as well. Fourth, we estimated work hours by the schedule in this hospital but did not measure every intern’s actual work hours. Quantifying and qualifying the stress during the internship could strengthen the relationship between stress and these hallucinations. Finally, since the vibration mode is associated with phantom vibration, whether the type or volumes of the ringing are also related to phantom ringing should be explored in order to strengthen the speculative mechanism of this hypothesis-guided selective search [33].

In conclusion, phantom vibration was more prevalent than phantom ringing syndrome before the internship year, and the incidence of both hallucinations significantly increased during the internship and the post-internship recovery period. This study suggested that phantom vibration and phantom ringing might be entities that are independent of anxiety or depression when evaluating stress-related experiences during medical internship training.
